# Androgens and Adult Neurogenesis in the Hippocampus

**DOI:** 10.1089/andro.2021.0016

**Published:** 2021-12-23

**Authors:** Samantha A. Blankers, Liisa A.M. Galea

**Affiliations:** ^1^ Graduate Program in Neuroscience, The University of British Columbia, Vancouver, Canada.; ^2^ Djavad Mowafaghian Centre for Brain Health, The University of British Columbia, Vancouver, Canada.; ^3^ Department of Psychology, The University of British Columbia, Vancouver, Canada.

**Keywords:** dentate gyrus, sex differences, aging, dihydrotestosterone, testosterone, cognition

## Abstract

Adult neurogenesis in the hippocampus is modulated by steroid hormones, including androgens, in male rodents. In this review, we summarize research showing that chronic exposure to androgens, such as testosterone and dihydrotestosterone, enhances the survival of new neurons in the dentate gyrus of male, but not female, rodents, via the androgen receptor. However, the neurogenesis promoting the effect of androgens in the dentate gyrus may be limited to younger adulthood as it is not evident in middle-aged male rodents. Although direct exposure to androgens in adult or middle age does not significantly influence neurogenesis in female rodents, the aromatase inhibitor letrozole enhances neurogenesis in the hippocampus of middle-aged female mice. Unlike other androgens, androgenic anabolic steroids reduce neurogenesis in the hippocampus of male rodents. Collectively, the research indicates that the ability of androgens to enhance hippocampal neurogenesis in adult rodents is dependent on dose, androgen type, sex, duration, and age. We discuss these findings and how androgens may be influencing neuroprotection, via neurogenesis in the hippocampus, in the context of health and disease.

## Introduction

Efforts to characterize the postnatal production of new neurons have been ongoing since the concept was first introduced through experiments by Joseph Altman in the 1960s.^1^ Although Altman's findings were initially dismissed by the scientific community, the notion of structural plasticity of dendritic spines in the adult brain gained acceptance with time^2–4^ (reviewed in Bosch and Hayashi^5^) and studies in songbirds confirmed that new cells produced postnatally were morphologically similar to neurons.^6,7^ Great progress has been made since these initial findings, through extensive research in neurogenic brain regions, including the hippocampus and olfactory bulb.^8–18^ Even in the early studies by Altman, it was suspected that androgens may be involved in the regulation of neurogenesis in the hippocampus.^1^ Indeed, decades later, it was noted that seasonal fluctuations in hippocampal neurogenesis coincide with changes in gonadal hormone levels in meadow voles^19^ and in black-capped chickadees^20^ of both sexes. Moreover, sex differences have been identified in the maturation and attrition of new neurons in the hippocampus of rats^21^ and in response to certain stimuli such as stress in rats^22,23^; for review see Ref.^24^ These findings warranted further investigation into the role of sex-steroid hormones in the regulation of hippocampal neurogenesis, including the sex-specific effects of estrogens and androgens. Thus, in this review, we discuss the stages and function of adult neurogenesis in the hippocampus, followed by the influence of androgens on different stages of hippocampal neurogenesis and in response to injury or disease. Within each section, we discuss sex differences in the influence of androgens, if known.

## Androgens

Androgens are sex steroid hormones produced in Leydig cells of the testes and thecal–interstitial cells of the ovaries, as well as in the zona reticularis of the adrenal glands in both males and females.^25,26^ In addition, androgens are produced in the brain itself, via either local production or *de novo* synthesis of steroids.^27–30^ It is also important to recognize that the systemic levels of androgens are associated with levels in the hippocampus^31^ and influence local and *de novo* production of androgens,^32^ although more studies are needed. Some of the widely studied androgens include testosterone, 5α-dihydrotestosterone (DHT), androstenedione, and dehydroepiandrosterone (DHEA) among others.^33^ Testosterone can be metabolized to other androgens or to estradiol, the most potent of the natural estrogens, and thus can exert its effects via androgen receptors (ARs) or estrogen receptors (ERs) depending on the availability of enzymes. Testosterone can be reduced to the potent androgen DHT via the enzyme 5α-reductase. DHT can be further metabolized to 5α-androstane-3α,17β-diol (3α-diol) and 5α-androstane-3β,17β-diol (3β-diol).^34^ Both 3α-diol and 3β-diol possess weak affinity for ARs,^35^ although 3β-diol displays preferential activity at ERβ.^36^ Testosterone can also be aromatized to estradiol,^34^ which binds to ERα, ERβ, and the G protein-coupled ER (GPER). The ARs and ERs are most often found in intracellular locations or on the nucleus, but they can also be located on the membrane.^37–39^ Ligand binding, when located in the intracellular compartment, causes the AR or ER to be transported to the nucleus, where they may influence transcription of target genes to elicit genomic effects that occur on a scale of hours to days.^40^ In addition, estradiol can bind to membrane-associated ERs such as GPER to induce rapid non-genomic effects through second-messenger signaling.^39^

The ERs are abundant in the hippocampus, with a high concentration of GPER in CA1, CA3, and the dentate gyrus^41^ and the highest density of ERα and ERβ in CA3 region compared with the other subregions of the hippocampus in male and female rats.^42,43^ On the other hand, ARs are not abundant in the dentate gyrus relative to the CA1 and CA3 regions of the hippocampus in both sexes.^44–48^. Indeed, the highest expression of ARs in the hippocampus is in the CA1 region in both sexes, which will vary by hormonal status (intact vs. gonadectomized).^44–48^ Studies have found that the density of ARs in the hippocampus increases with age, as serum testosterone levels decline.^47,49^ Sex differences in AR expression have been detected, as the density of AR in the CA1 region of males is greater than that of the CA1 region of females^48^ but this is not seen in all studies,^47^ likely due to differences in whether gonadectomized versus intact rats were used. Indeed, intact males had higher AR expression in the CA1 region compared with intact females^49^ but there are no significant sex differences in AR expression when comparing gonadectomized rats.^47^ In addition, the gene encoding the AR protein contains a polymorphic cytosine-adenine-guanine (CAG) microsatellite of variable lengths ranging from 6 to 39 repeats at the N-terminal transactivation domain.^50^ The functionality of ARs depends on the number of CAG repeats, as transactivation of AR decreases with CAG repeat expansion.^51^ Therefore, androgens act through ERs or ARs that are expressed in the hippocampus and influenced by several factors such as age, sex, and genetics.

## Adult Neurogenesis

**FIG. 1.**
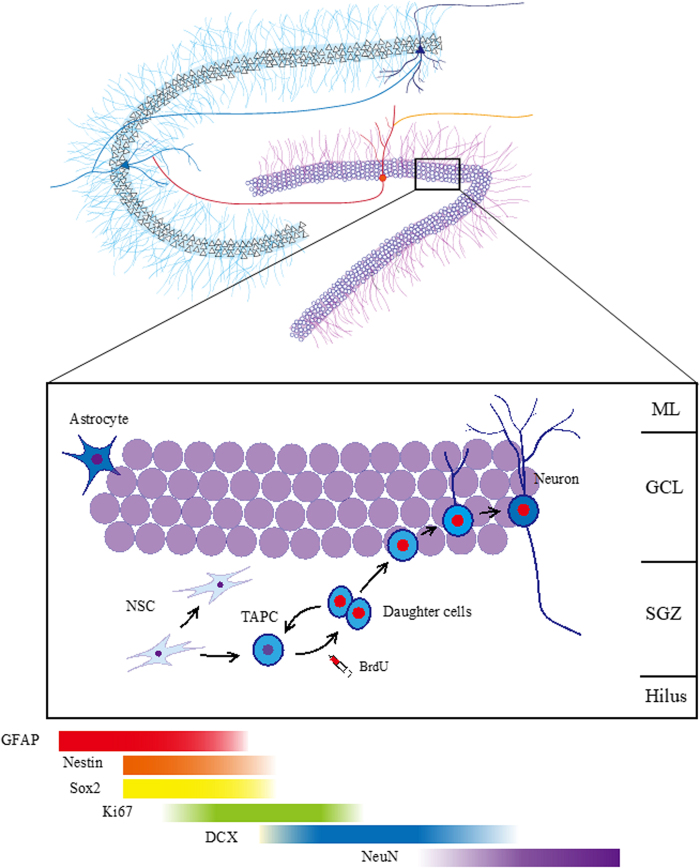
Schematic image of a coronal section of the rodent hippocampus and stages of adult neurogenesis. An NSC can give rise to a TAPC, which is divided into two NPCs that migrate in the GCL of the dentate gyrus, differentiate into neurons, and send dendrites to the ML.^52^ The NSCs express the glia cell marker, GFAP, and NSC markers, Nestin, and Sox2.^52^ Ki67 is a nuclear protein that is expressed during all active phases of the cell cycle and thus is a marker of cell proliferation.^53^ DCX is a microtubule-associated protein expressed in the cytoplasm of dividing NPC and immature neurons.^54^ NeuN protein is expressed in new neurons beginning about 1 week after mitosis in rats and 2 weeks after mitosis in mice and is used as a nuclear marker for mature neurons.^55^ BrdU is a DNA synthesis marker that is incorporated into cells that are actively synthesized DNA within the 2 h after injection (and in effect time stamps when the cell was produced). Depending on the length of time between injection and perfusion, BrdU-immunoreactive cells will label cell proliferation or the survival of new cells. Zif268 is an immediate early gene and will indicate whether a cell has been recently active (action potential). Reprinted with permission from Yagi et al.^21^ BrdU, 5-bromo-2-deoxyuridine; DCX, doublecortin; GCL, granular cell layer; GFAP, glial fibrillary acidic protein; ML, molecular layer; NeuN, neuronal nuclei; NPC, neural progenitor cells; NSC, neural stem cell; SGZ, subgranular zone; TAPC, transient amplifying progenitor cell.

Neurogenesis is defined as the creation and functional integration of new neurons produced from neural stem/progenitor cells. There are several stages of neurogenesis that include cell proliferation, migration, differentiation, and survival of newly generated neurons (Fig. 1). Various internal and external factors have been identified as regulators of neurogenesis in each one of these stages; therefore, it is critical to be aware of the stage of neurogenesis being evaluated within an individual experiment. There are many methods available for the detection of newly formed neurons, each with their own strengths and limitations.^53*,*56,57^ Endogenous proteins, such as Ki67, are expressed during all stages of the cell cycle except G_0_ and are used to measure cell proliferation.^53^ Doublecortin (DCX), another endogenous protein, is used to measure the presence of immature neurons, as it is expressed during proliferation until approximately day 21 in rats.^54^ Exogenous DNA-markers, such as ^3^H-thymidine, or synthetic nucleosides, such as 5-bromo-2-deoxyuridine (BrdU), may be used to monitor the production and survival of new neurons depending on the time between injection and perfusion. These markers are incorporated during DNA synthesis and may be visualized postmortem to detect newly generated cells in a region of interest.^58,59^ The timing between administration of DNA synthesis markers and perfusion of the animal is significant, as different time spans will measure different stages of neurogenesis. If animals are perfused 24 h or less after BrdU administration, this will be a measure of cell proliferation, whereas a span of >24 h will measure survival of the daughter cells.^56,60^ Further, the timing of experimental manipulation in relation to BrdU administration is important, as this will capture survival of new neurons either dependent or independent of the influence of cell proliferation; for review see Refs.^56,61^ It is noteworthy that endogenous markers of mature neurons must be used in combination with thymidine analogues to determine whether newly formed cells are new neurons; therefore, the co-expression of BrdU with endogenous markers such as neuronal nuclei is often used to detect new neurons versus the use of glial fibrillary acidic protein used for the detection of new glial cells.^62^

As expected, neurogenesis occurs at a high level during development and diminishes in adulthood, and this has been observed across a wide variety of species, including primates^8,9^ and rodents.^10,11^ Under normal conditions, neurogenesis occurs in two distinct regions of the brain: the subventricular zone (SVZ), the lining of the lateral ventricles and the subgranular zone (SGZ) of the hippocampus. The SVZ contains mostly multipotent neural stem cells (NSCs), whereas the proliferative precursor cells of the SGZ were previously identified as mainly neural progenitor cells (NPC).^63,64^ However, studies have revealed the presence of multipotent NSCs in the SGZ,^65–67^ which ultimately give rise to NPC^68^ that proliferate and migrate to the granular layer where most will differentiate into granule cells.^1,69^ Multipotent NSCs have the capacity for self-renewal and can give rise to multiple types of mature neural cells.^70^ The NPC are distinct, as they have a limited capacity for self-renewal and usually differentiate into one specific type of neural cell.^70^ The NPC originating in the SGZ proliferate and migrate to the granular layer, where most will differentiate into granule cells.^1,69^ Intriguingly, a few studies suggest that there is little decline in the number of new immature neurons throughout adulthood in humans.^16–18^ Evidence for neurogenesis in the hippocampus of humans has been demonstrated in a multitude of studies using a variety of techniques.^12–18^ Thus, these studies support the consensus that neurogenesis in the human hippocampus is evident throughout adult life, although this claim is not entirely undisputed.^71,72^ This review will focus on the influence of androgens in hippocampal neurogenesis, and the reader is directed to other reviews on hormones and the SVZ.^73,74^

## Function of Adult Hippocampal Neurogenesis

It is one thing to produce new neurons in the adult brain, but do these new neurons form meaningful connections to alter the function of the hippocampus? It is important to recognize that a new neuron could make aberrant connections that might interfere with the normal function of the dentate gyrus.^75,76^ Ectopic new neurons can interfere with normal activity of the dentate gyrus, as seen with hippocampal neurogenesis after seizures in animal models of temporal lobe epilepsy.^75,77^ Populations of these new neurons form aberrant axon projections that are characteristic of those seen in postmortem humans with temporal lobe epilepsy,^77,78^ and this abnormal cytoarchitecture may result in long-term alterations of hippocampal circuitry.^75^ Indeed, seizure-induced increases in neurogenesis is commensurate with reduced memory, and when neurogenesis is reduced in response to seizures, memory is improved.^75^ On the other hand, voluntary wheel-running increases both hippocampal neurogenesis and performance on several new memory tasks in mice.^79–81^ Jakubs et al. used whole cell-patch clamp recordings to compare the properties of new neurons generated on exposure to either running or induced seizures in rats. Both running and seizures will increase neurogenesis in the hippocampus, but new neurons generated in runners demonstrated higher excitatory synaptic drive and lower inhibitory synaptic drive compared with new neurons generated with seizures.^76^ Together, these findings demonstrate that new neurons may have distinct functional properties that are dependent on the circumstances of which neurogenesis was induced; thus, increased neurogenesis is not always beneficial to the individual if atypical integration occurs; for review see Refs.^82–84^

New neurons appropriately integrated into the hippocampal circuit are believed to influence and support brain functioning in a variety of ways.^24,52,61,68,85^ The hippocampus is critical in forming contextually rich memories^86^ due to the neuronal characteristics of the dentate gyrus. That is, discrete representations of memory are possible because neurons of the dentate gyrus are able to discriminate between small differences in cortical input patterns.^87^ Briefly, adult hippocampal neurogenesis is critical in discrimination between highly similar situations,^88–92^ a phenomenon known as pattern separation.^93^ Other studies have shown that hippocampal neurogenesis is critical for context encoding in spatial discrimination tasks and contextual/trace fear conditioning^89,94–97^ in both male and female mice; for review see Refs.^98,99^ Functional differences exist between the dorsal and ventral hippocampus due in part to their distinct connections to extra-hippocampal structures, receptor density patterns, and gene expression patterns.^100,101^ The dorsal hippocampus is responsible for cognitive processing whereas the ventral hippocampus regulates emotional processing, including mood and stress.^102^ It has been proposed that hippocampal neurogenesis, particularly in the ventral hippocampus, is implicated in this emotional regulation.^103^ Animal models of depression in male and female rodents decrease neurogenesis in the ventral dentate gyrus^104^ and chronic, but not acute, treatment with pharmacological antidepressants restores neurogenesis in the dentate gyrus of male and female rodents.^24,105,106^ In primates, the anterior hippocampus is akin to the ventral hippocampus of rodents, and humans with major depressive disorder show decreased cell proliferation^107^ which is increased with antidepressant exposure^107^ in the anterior hippocampus, dependent on factors such as age and sex.^108^ Further, Surget et al.^109^ found that the antidepressant fluoxetine was not effective in restoring a dysregulated hippocampal-driven response to chronic stress unless an intact neurogenic system was present in the ventral hippocampus of male mice. Therefore, numerous lines of evidence suggest that neurogenesis in adulthood influences various aspects of brain function, including mood, stress, and cognition, dependent on the brain region in which new neurons are integrating.

## Androgens and Adult Hippocampal Neurogenesis

In the following sections, we discuss the influence of androgen manipulations on hippocampal neurogenesis in males and females across the adult lifespan and in response to injury or disease.

## Castration and Adult Hippocampal Neurogenesis

Reproductive status influences neurogenesis in the hippocampus of male rodents.^110,111^ Reproductively active male meadow voles had increased survival of new neurons compared with reproductively inactive male meadow voles.^110^ Castrated adult rats and mice show decreased survival of new neurons or fewer immature neurons in the dentate gyrus compared with intact males.^111–114^ On the other hand, castration before adolescence does not have the same effect on hippocampal neurogenesis as it increased neurogenesis in the hippocampus of male rhesus macaque monkeys^115^ and had no significant influence on hippocampal neurogenesis in male rats.^116^ These studies collectively indicate that the timing of castration during the lifespan matters for the influence on hippocampal neurogenesis, with adult castration decreasing but adolescent castration either increasing or not affecting neurogenesis in the hippocampus.

## Androgens and Adult Hippocampal Neurogenesis in Males

Chronic exposure to androgens generally upregulates neurogenesis in the hippocampus of male, but not female, rodents via enhancement of new neuron survival in a dose-dependent manner.^47,111^ Thirty days of testosterone or DHT exposure enhances neurogenesis by promoting the survival of new neurons in male rodents.^111^ Interestingly, this effect is not seen with shorter testosterone replacement schedules of 15–21 days,^106,117^ indicating that 30 days of testosterone replacement is necessary to observe increased survival of new neurons in the hippocampus. The influence of testosterone to promote neurogenesis in the hippocampus may be independent of any effects on cell proliferation, as 21 days of testosterone did not influence cell proliferation.^106^ However, long-term but not short-term castration in adulthood decreases cell proliferation in rats,^111,112^ suggesting that there may be an influence of longer-term androgen exposure on cell proliferation in the dentate gyrus.

Several lines of evidence support the notion that testosterone exerts its influence on hippocampal neurogenesis by interacting with the AR. First, treatment with estradiol has no significant effect on the survival of new neurons in adult males,^111^ indicating that ER activation is not responsible for neurogenic effects of androgens in males. On the other hand, DHT, which directly stimulates the AR, increased the survival of new neurons, similar to testosterone.^44,111^ The neurogenesis-promoting effect of DHT was blocked in the presence of flutamide, a competitive AR antagonist.^44^ Moreover, chronic testosterone had no effect on the survival of new neurons in rats with a testicular feminization mutation, which lack a functional AR,^44^ indicating that androgens influence neurogenesis through an AR-mediated mechanism. However, considering that testosterone and DHT increase neurogenesis by promoting the survival of new neurons, it is a matter of curiosity that ARs are not located on immature neurons (DCX-expressing cells) in male rats or mice in the dentate gyrus.^44,114^ On the other hand, ARs are expressed in the CA3 region of the hippocampus,^45–47^ which is the axonal projection site of newly formed neurons.^118,119^ Androgens, via the AR, rapidly increase thorny excrescences in the CA3 region, which are the post-synaptic regions of the mossy fibers.^120^ Therefore, it is possible that the small number of ARs located in the dentate gyrus and/or ARs located in the terminal region for granule cells in the CA3 region are responsible, at least in part, for the AR-dependent promotion of neurogenesis in the hippocampus.

## Androgens and Adult Hippocampal Neurogenesis in Females

**FIG. 2.**
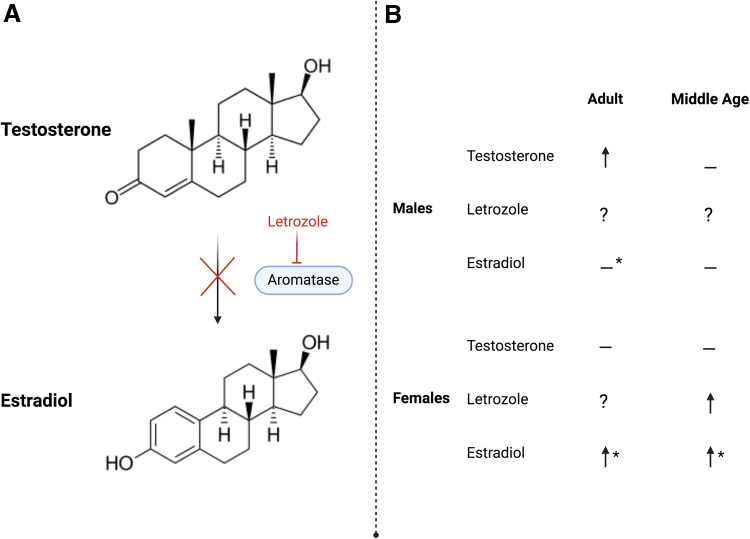
**(A)** Aromatase inhibition with letrozole. In the final stage of estrogen synthesis, the aromatase inhibitor letrozole prevents the aromatization of testosterone into estradiol. **(B)** Testosterone, letrozole, and estradiol effects on survival of new neurons in the hippocampus of adult roents. In males, testosterone, but not estradiol, treatment increases the survival of new neurons in adults, although no significant effect is seen in middle age. The influence of letrozole has yet to be studied in males. In females, testosterone does not influence cell survival in either adulthood or middle age. Letrozole increases new neuron survival in middle-aged females, although studies in adults have yet to be completed. Lastly, estradiol increases the survival of new neurons in both adult and middle-aged females, although the paradigm used in the experiment influences the resulting effects on new neuron survival as indicated with an asterisk (*). Figure created using Biorender.com.

Although androgens promote neurogenesis in males, 30 days of testosterone or DHT treatment do not affect neurogenesis in young adult or middle-aged female rats.^47^ Although these findings suggest that androgens do not support neurogenesis in females, Chaiton et al.^121^ found that chronic treatment with letrozole, an aromatase inhibitor, increased neurogenesis (cell proliferation and density of immature neurons) in the hippocampus of middle-aged female mice. As letrozole inhibits the conversion of androgens into estradiol (Fig. 2), this result implies that the increase in neurogenesis may be related to the increase in androgens induced by letrozole, and/or the decrease in estradiol synthesis. Although letrozole increased the number of doublecortin cells (immature neurons) of middle-aged females, it suppresses the cell proliferation of cultured neurons from postnatal day 5 female rats,^122^ indicating age differences in the influence of letrozole on neurogenesis in the hippocampus.

In adult females, the influence of estradiol on adult hippocampal neurogenesis is complex, as it is dependent on dose, sex, age, reproductive experience, and timing of estradiol exposure^123–127^; for review see Ref.^24^ As mentioned earlier, the survival of new neurons is enhanced by chronic exposure to testosterone and DHT, but not estradiol, in adult male rats.^111^ However, other studies show that brief exposure to estradiol can increase neurogenesis in male meadow voles when administered during the time when new neurons are extending their axons.^125^ Together, these results demonstrate that sex hormone-mediated regulation of hippocampal neurogenesis is distinct in males compared with females, with more neurogenic effects of androgens in males and more neurogenic effects of estrogens in females.

## Age Effects of Androgens and Hippocampal Neurogenesis

Age is an additional factor that influences the effects of androgens on neurogenesis in males. Chronic DHT treatment increased new neuron survival in young, but not middle-aged, adult gonadectomized male rats.^47^ Consistent with this, Moser et al.^128^ found that 12 weeks of testosterone failed to increase neurogenesis (DCX-expressing cells) in both intact middle-aged and aged male rats. These findings are in contrast with the increased neurogenesis observed in young adult males with chronic testosterone or DHT,^111^ suggesting that aging influences the ability of the dentate gyrus to respond to the pro-neurogenic effects of androgen signaling in male rats. This is a matter of curiosity, as ARs are evident in the dentate gyrus of middle-aged rodents, potentially at even higher levels than in young adults.^47,49^ Although AR levels may be higher in aged males compared with young males,^47,49^ it is possible that AR functionality decreases with age. Further, it is noteworthy that long-term castration resulted in loss of granule neurons in the dentate gyrus of adult male rats, which can be ameliorated by early but not late chronic DHT treatment,^129^ indicating a critical window for the neuroplasticity-promoting effect of DHT. Whether or not this critical window exists with respect to DHT's ability to increase adult hippocampal neurogenesis has not yet been studied. However, it is evident that androgens are not promoting neurogenesis in intact or gonadectomized middle-aged animals, but it remains to be determined whether this has to do with dose, critical window, or the functionality of AR with aging.

Here, we concentrate our discussion on adult neurogenesis in the hippocampus, but it is important to consider that androgen exposure during gestation or the early postpartum period influences neurogenesis of the developing offspring in both sexes.^130–133^ Briefly, mothers exposed to a hyperandrogenic environment during gestation produced offspring with decreased survival of new neurons in the dentate gyrus of both male and female rats.^130^ On the other hand, there is evidence that androgens promote the survival of new neurons during the postpartum period in rats of both sexes.^132,133^ Thus, there are likely distinct mechanisms by which androgens influence neurogenesis during gestation and early development.

## Effects of DHEA and Androstenedione on Hippocampal Neurogenesis

Although the majority of studies on androgens and neurogenesis have focused on testosterone and DHT, there is some evidence for the pro-neurogenic effects of DHEA.^134–137^ DHEA has distinct cellular effects from other androgens, such as DHT and testosterone, as DHEA preferentially activates ERβ while also demonstrating affinity for ERα and AR^138–140^ along with other nuclear and membrane-bound receptors (reviewed in Prough et al.^141^). The NSCs derived from the human male fetal cortex demonstrated increased cell survival with DHEA treatment compared with controls.^137^ In adult and middle-aged male rats, DHEA increases the survival of new neurons after repeated or chronic treatment.^134,135^ Short-term treatment (12 days) with DHEA increased the number of new neurons as well as cell proliferation in the dentate gyrus of adult male rats, and pretreatment with DHEA was protective against corticosterone injections, which inhibit neurogenesis.^135^ The same study found that pretreatment with androstenedione had no influence on the inhibition of neurogenesis seen with corticosterone administration^135^ but did not evaluate androstenedione alone. In adult male songbirds, although DHEA increased immature neurons (DCX-expressing cells) in the HVC, a brain region involved in song production, it did not do so in the hippocampus.^136^ Thus, DHEA promotes the survival of new neurons in multiple species, increasing cell proliferation and protecting against stress in adult male rats specifically.

## Androgenic Anabolic Steroids and Hippocampal Neurogenesis

Other compounds of interest include androgenic anabolic steroids (AAS) such as the testosterone analogue 19-nortestosterone, also known as nandrolone. Excessive use of AAS has been linked to mood disturbances^142^ such as mania and major depression in humans. On the cellular level, nandrolone elicits its effects in the brain by binding to the AR and repeated administration causes AR upregulation.^143^ A study utilizing rat NSCs in culture and in the dentate gyrus revealed that the administration of nandrolone for 5 days decreased the number of new neurons detected in the dentate gyrus through an AR-mediated mechanism.^144^ Another study showed that chronic (28 days) treatment with supraphysiological doses of nandrolone eliminated the strength training-induced enhancement of cell proliferation in the dentate gyrus of adult male Wistar rats.^145^ These studies indicate that AAS reduce hippocampal neurogenesis in a manner distinct from that of testosterone and DHT, which may be linked to the supraphysiological doses used or the molecular differences between these androgens.

## Androgens and Adult Hippocampal Neurogenesis: Implications for Health and Disease

Androgen treatments in clinical disease may afford neuroprotection via their influence on neurogenesis in the hippocampus.^146^ Low levels of androgens are associated with an increased risk for stroke, cerebrovascular disease, major depressive disorders, and dementia.^147–150^ Androgens have been used to treat these diseases with some success, which depends on a variety of factors.^146^ In addition, androgens play a role in neuroprotective factors that boost neurogenesis, such as exercise. Exercise-induced neurogenesis is inhibited in the presence of the AR antagonist flutamide, and mild exercise stimulates DHT production in male rats, which, in turn, enhances neurogenesis in the hippocampus.^151^ Thus, androgens may be involved in some of the neurogenic responses to environmental factors and demonstrate neuroprotective effects against injury and disease.

Ischemic stroke is associated with impairments in memory along with increased neurogenesis,^152^ but as with seizure-induced neurogenesis in the hippocampus^76^ these new neurons form aberrant connections.^152^ Indeed, the reduction of post-stroke neurogenesis aids in the retention of memory formation. Intriguingly, although castration and flutamide did not alter hippocampal neurogenesis 1 week after stroke, supraphysiological doses of testosterone and DHT reduced post-stroke hippocampal neurogenesis in the same study.^153^ Thus, one way that androgens may afford protection in the face of stroke is to reduce ischemia-induced neurogenesis in the hippocampus that may be related to aberrant connections tied to poorer memory outcomes.

Androgens play an important role in reducing depression in hypogonadal males, as meta-analyses demonstrate that testosterone treatment in hypogonadal men effectively reduces the symptoms of depression.^146,154,155^ Castration increased the susceptibility to depressive-like endophenotypes in the face of chronic unpredictable stress,^112^ which was commensurate with reduced neurogenesis in the hippocampus. However, testosterone treatment did not rescue the decrease in cell proliferation observed under social isolation stress in castrated males.^117,156^ Interestingly, testosterone and the tricyclic antidepressant imipramine increased cell proliferation in the hippocampus more than antidepressant treatment alone in response to social isolation stress, an effect observed in males but not females.^156^ Further, testosterone in conjunction with imipramine increased polysialylated neuronal cell adhesion molecule (PSA-NCAM)-ir cells in the dentate gyrus, but not neurogenesis *per se*, after exposure to chronic unpredictable stress.^106^ In another model of depression using olfactory bulbectomy, the androgen DHEA prevented depressive-like behavior and increased the number of 1-week-old new neurons in response to bulbectomy in adult male mice.^157^ DHEA has antidepressant effects^157^ and works synergistically with fluoxetine, a selective serotonin reuptake inhibitor, causing an otherwise ineffective dose of fluoxetine to increase cell proliferation in the dentate gyrus of adult male rats.^158^ Thus, collectively these data demonstrate that androgens can rescue depressive-like behavior and facilitate antidepressant efficacy, perhaps via its ability to stimulate neurogenesis.

In epilepsy, androgens can influence seizure susceptibility in males.^159–163^ In males with epilepsy, lower levels of free testosterone and sexual dysfunction can be observed^164^ but the directionality of this effect and whether treatments for epilepsy compound this relationship are not clear.^164,165^ However, in animal studies, castrated rodents are more susceptible to pentylenetetrazol, picrotoxin, and perforant pathway stimulation-induced seizures compared with intact males, and testosterone administration decreases seizure activity in castrated males.^159–161^ There is also evidence to suggest that the testosterone metabolite 3α-diol is protective against gamma-aminobutyric acid (GABA)_A_ receptor antagonist-induced seizures in male mice,^162,163^ which is interesting as 3α-diol has a weak affinity for ARs.^41^ However, the relationship between sex steroid hormones and epilepsy has proven difficult to discern as various factors such as experimental conditions and biological variability can greatly influence study outcomes (reviewed in Scharfman and MacLusky^166^). Although the mechanisms of androgens' influence on seizure activity are not yet clear, it should be recalled that seizures induce aberrant hippocampal neurogenesis^75,77^; thus, it is plausible that androgens may be protective via their effects on neurogenesis in the dentate gyrus to reduce the aberrant connectivity of new neurons.

Finally, androgens may play a protective role in neurodegenerative diseases such as Alzheimer's disease (AD); for review see Ref.^167^ Low levels of free testosterone may be a risk factor for AD in males,^168–170^ and males with AD exhibit lower levels of testosterone in the periphery and in the brain compared with age-matched controls.^149,171,172^ Two copies of the *APOE*e4 allele confer a greater risk to develop sporadic late-onset AD^173,174^ and female *APOE*e4 carriers have disproportionately increased phosphorylated Tau (p-Tau), a neuropathological feature of AD, compared with male carriers.^175^ Further, low serum testosterone in both males and females corresponded to increased p-Tau,^176^ suggesting that the low levels of testosterone in both sexes may confer a greater risk of neuropathology related to AD. Testosterone treatment given to males with AD or mild cognitive impairment, a prodromal state to AD, increased scores on certain forms of memory, including spatial memory.^177^ Thus, androgens may improve certain types of memory in both sexes with age and given that the hippocampus undergoes early degeneration with AD, it is possible that androgens may promote cognition during aging and in AD, via its effects on hippocampal neurogenesis. Intriguingly, neurogenesis in the hippocampus is decreased in people with AD when compared with healthy aging controls.^12,13,178^ In addition, low testosterone coupled with APOEe4 genotype was related to lower hippocampal volume^179^ and poorer verbal episodic memory.^180^ Thus, several studies suggest that the androgens are related to hippocampal structure and function and more work is needed to explore the relationship between androgens and neurogenesis in AD and aging.

## Conclusion

Chronic but not acute exposure to a variety of androgens generally increases neurogenesis in the hippocampus of adult males, an effect not seen in females or in middle-aged males. Despite the progress in this field, it is currently not known how or where ARs work to promote neurogenesis in the dentate gyrus with chronic testosterone or DHT, and whether androgen-induced new neurons are contributing to the function of the dentate gyrus. The addition of new neurons in the dentate gyrus has functional implications, but improper integration of new neurons may be deleterious to the function of these circuits. In addition, environmental influences such as central nervous system injury, disease, stress, and exercise all play a role in the neurogenic response to androgens. Sex differences observed in the neurogenic response to sex steroid hormones indicate that males and females have distinct hormonal mechanisms for the control of neurogenesis, and further work should explore the benefits of androgens to influence neurogenesis in males and possibly females, particularly in relation to health and disease.

## Abbreviations Used

3α-diol: 5α-androstane-3α,17β-diol
3β-diol: 5α-androstane-3β,17β-diol
AAS: androgenic anabolic steroids
AD: Alzheimer's disease
ARs: androgen receptors
BrdU: 5-bromo-2-deoxyuridine
CAG: cytosine-adenine-guanine
DCX: doublecortin
DHT: 5α-dihydrotestosterone
DHEA: dehydroepiandrosterone
ERs: estrogen receptors
GCL: granular cell layer
GFAP: glial fibrillary acidic protein
GPER: G protein-coupled ER
ML: molecular layer
NeuN: neuronal nuclei
NPC: neural progenitor cells
NSC: neural stem cell
NSERC: Natural Sciences and Engineering Research Council of Canada
p-Tau: phosphorylated Tau
SGZ: subgranular zone
SVZ: subventricular zone
TAPC: transient amplifying progenitor cell
